# Clinical and Genetic Spectra of Inherited Liver Disease in Children in China

**DOI:** 10.3389/fped.2021.631620

**Published:** 2021-03-04

**Authors:** Youhong Fang, Jindan Yu, Jingan Lou, Kerong Peng, Hong Zhao, Jie Chen

**Affiliations:** National Clinical Research Center for Child Health, Department of Gastroenterology, The Children's Hospital, Zhejiang University School of Medicine, Hangzhou, China

**Keywords:** genotype, phenotype, next-generation sequencing, child, inherited liver disease

## Abstract

**Background:** Children presenting with chronic liver disease or acute liver failure often have an underlying genetic disorder. The aim of this study was to analyze the clinical and genetic spectra of inherited liver disease in children in a tertiary hospital.

**Methods:** A total of 172 patients were classified into three groups according to their clinical presentation: cholestasis (Group A), liver enzyme elevation (Group B), and hepato/splenomegaly (Group C). Next-generation sequencing (NGS) was performed on all patients recruited in this study. The genotypic and phenotypic spectra of disease in these patients were reviewed.

**Results:** The median age at enrollment of the 172 patients was 12.0 months (IQR: 4.9, 42.5 months), with 52.3% males and 47.7% females. The overall diagnostic rate was 55.8% (96/172) in this group. The diagnostic rates of whole-exome sequencing (WES) and targeted gene panel sequencing (TGPS) were 47.2% and 62.0%, respectively (no significant difference, *p* = 0.054). We identified 25 genes related to different phenotypes, including 46 novel disease-related pathogenic mutations. The diagnostic rates in the three groups were 46.0% (29/63), 48.6% (34/70), and 84.6% (33/39). *ATP7B, SLC25A13*, and *G6PC* were the top three genes related to monogenic liver disease in this study.

**Conclusion:** WES and TGPS show similar diagnostic rates in the diagnosis of monogenic liver disease. NGS has an important role in the diagnosis of monogenetic liver disease and can provide more precise medical treatment and predict the prognosis of these diseases.

## Introduction

Children with chronic liver disease or acute liver failure often have underlying genetic disorders that not only caused by liver-based diseases but also systemic diseases (1). Liver diseases caused by gene mutations are defined as inherited liver diseases or monogenic liver diseases. These diseases often present with overlapping phenotypes and lack specific laboratory testing indicators, which may lead to delays in diagnosis and improper treatment. The diagnosis of inherited liver diseases relies on extensive biochemical and histologic studies, which are often expensive, time consuming, and affected by the availability of specific biochemical tests and the experience of the researchers. In recent years, next-generation sequencing (NGS) has been widely used in clinical activities, especially using targeted gene panel sequencing (TGPS) and whole-exome sequencing (WES), to detect causative mutations of monogenic diseases. Studying the clinical and genetic spectra of these diseases can lead to a better understanding of the relationship between them. The precise disease type can be classified according to the genetic results, and a timely diagnosis promotes prompt specific treatment and a better outcome. To the best of our knowledge, no such large study has focused on the genotypes and phenotypes of pediatric inherited liver diseases. The aim of this study was to analyze the clinical and genetic spectra of inherited liver diseases in children by using WES and TGPS, and to compare the diagnostic yield of these two methods in a tertiary hospital in China.

## Materials and Methods

### Patient Samples

Patients aged from one month to 16 years old were retrospectively recruited from the Gastroenterology Department, Children's Hospital, Zhejiang University School of Medicine, from May 2005 to October 2019. They were classified based on their clinical presentation as follows: cholestasis (Group A), liver enzyme elevation (Group B), and hepato/splenomegaly (Group C). Cholestasis was defined in the presence of a serum conjugated bilirubin level >1 mg/dL (2). Liver enzyme elevation was defined as isolated transaminase elevation and combined elevation of alkaline phosphatase (ALP) and gamma-glutamyl transpeptidase (GGT). The diagnostic algorithm was as follows: first, liver diseases caused by biliary atresia, infection, drug hepatotoxicity, autoimmune hepatitis (AIH), and alcohol liver disease were excluded according to the history, physical examination and initial workup for viruses, bacteria and autoantibodies associated with autoimmune diseases. Second, patients in all three groups underwent liver function tests, including tests to determine the levels of the liver enzymes alanine aminotransferase (ALT), aspartate aminotransferase (AST), GGT, and ALP, serum albumin level, lipid metabolism profile, coagulation profile, serum/urea tandem mass spectrometry analysis, blood ammonia level, blood lactic acid level, and blood gas analysis. Patients with suspected genetic diseases such as Alagille syndrome (AS), Wilson disease, glycogen storage disease (GSD), progressive familial intrahepatic cholestasis (PFIC), bile acid metabolic disease, and citrin deficiency were studied via TGPS or WES, and patients without suspected genetic diseases were studied via WES.

This study was approved by the Ethical Committees of Children's Hospital, Zhejiang University School of Medicine (Identifier: 2019-IRB-167). Written consent for participation was collected from the parents of some patients.

### Next-Generation Sequencing

The processes of WES and TGPS have been described previously (3). The candidate causal mutations were then confirmed by Sanger sequencing, and cosegregation analyses among the families were also conducted. The processes of Sanger sequencing have also been described previously (3).

### Variant Analysis

Interpretation of variants was based on recommended standards from the American College of Medical Genetics and Genomics (4), and all variants were categorized as pathogenic, likely pathogenic, variants of unknown significance, likely benign or benign. The variants classified as pathogenic or likely pathogenic related to disease were defined as pathogenic variants.

### Statistical Analysis

Categorical variables are presented as numbers (percentages). Continuous variables with a normal distribution are presented as the mean ± SD or as the median and interquartile range (IQR). The normality test was performed by the Shapiro-Wilk test. The difference in diagnostic yield between WES and TGPS was assessed by the chi-square test. The significance level was 0.05. All statistical analyses were conducted with SPSS 22.0 statistical software (SPSS Inc., IBM Corp., Armonk, NY, United States).

## Results

### Demographic Features of the Patients

In all, 172 patients were enrolled in this study. There were 90 (52.3%) males and 82 (47.7%) females. A total of 93.8% of the patients were within six years of age at the time of enrollment, and 56.3% of the patients were within two years of age. The median age at enrollment was 12.0 months (IQR: 4.9, 42.5 months). No consanguineous marriage was found in our cohort. Detailed information on the patients in the three groups is listed in Table 1. Only one patient died, with a mortality rate of 0.6%, and two patients underwent liver transplantation during follow-up.

**Table 1:** The basic information of the three groups of patients.

	**Group A, *n* = 63**	**Group B, *n* = 70**	**Group C, *n* = 39**	**Total, *n* = 172**
Male/Female, *n*	35/28	35/35	20/19	172
Age (median, IQR), (month)	4.7(2.3, 10.0)	36.0(12.0, 51.0)	13.0(11.6, 28.0)	12.0(4.9, 41.7)
Monogenic liver disease, *n*	29	34	33	96
Mortality, *n*	1	0	0	0

*Group A, cholestasis; Group B, liver enzyme elevation; Group C, hepato/splenomegaly*.

### Diagnostic Yield of Next-Generation Sequencing

**Figure 1:**
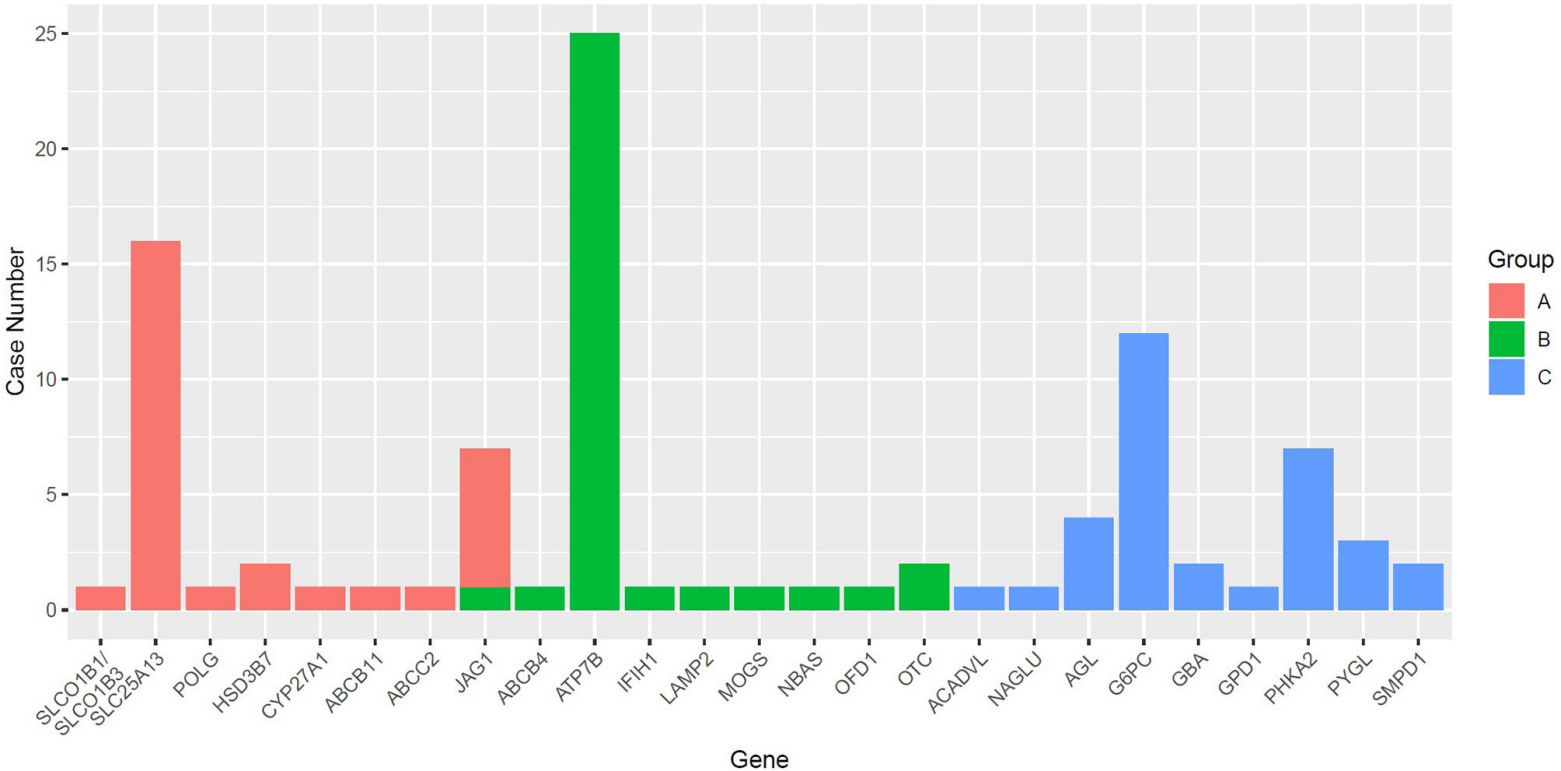
The number of different genes identified among different groups. *SLC25A13, ATP7B*, and *G6PC* were the most frequently identified genes in Group A, Group B, and Group C.

In this study, WES and TGPS were performed on 72 and 100 patients, respectively. Monogenic liver diseases were confirmed in 96/172 (55.8%) patients, including 34 cases identified by WES and 62 cases by TGPS. The diagnostic rate was not significantly different between WES and TGPS (*p* = 0.054). There were 25 genes related to genetic liver disease in this study cohort. Eight genes were identified in Group A, and nine genes each were identified in both Group B and Group C. The gene distributions and numbers of identified gene mutations with different ages and different phenotypes are shown in Figure 1. *ATP7B, SLC25A13*, and *G6PC* were the top three genes related to monogenic liver disease in this study. Moreover, among the identified monogenic liver disease patients, 22 patients carried homozygous mutations, 56 patients harbored compound heterozygous mutations, and 18 patients had heterozygous mutations, which included 10 hemizygous mutations and eight autosomal dominant mutations. Twenty-three patients carried a heterozygous pathogenic variation, and no pathogenic variant was found in 53 patients. Among these pathogenic variants, 46 variants were identified as novel (Supplementary Table 1).

### Phenotypes and Genotypes in the Three Subgroups

#### Patients With Cholestasis

The diagnostic rate in the cholestatic liver disease group was 46.0% (29/63). The most frequent disease was citrin deficiency, which accounted for 25.4% (16/63) of the cases. Seven patients had homogeneous mutations, and nine patients had compound heterozygous mutations in the *SLC25A13* gene (NM_014251). Mutation c.851_854del4 p. Met285Profs^*^2 was the predominant variant identified in this cohort. A total of 46.9% of variants were c.851_854del4 p. Met285Profs^*^2, which was lower than previous reports ranging from 63.7% to 80.6% in the Chinese population (5, 6). Two novel mutations, c.475C>T p. Q159^*^ and c.401T>A p. V134E, were not previously reported. Cholestasis was resolved in all patients within six months after starting dietary treatment. The second most frequent disease was Alagille syndrome (AS), which is caused by a *JAG1* mutation. Among seven *JAG1* mutations, four were de novo mutations. We identified five new variants: c.887-2A>G splicing, c.694+1G>A splicing, c.487C>A p. P163T, c.2850delT p.Asp951Thrfs^*^19 and c.35_45delGCCCCCTAAGC p. R12Pfs^*^57. One patient with a heterozygous mutation in *ABCB11* underwent liver transplantation, and all other patients presented with cholestatic hepatitis. Jaundice resolved during follow-up, and no patients developed hepatic cirrhosis. Thirty-three patients had negative findings by NGS.

Four patients were clinically diagnosed with citrin deficiency, and NGS did not confirm the diagnosis. Three patients with low GGT levels were suspected to have PFIC; however, NGS detected heterozygous mutations in the *TJP2* and *ABCB11* genes in two patients and negative mutations in one patient. The diagnosis in 19.0% (12/63) of the patients in this group could not be made based on initial laboratory workup and was finally diagnosed by NGS; these patients included one with an *SLC25A13* mutation, one with a *POLG* mutation, five with a *JAG1* mutation, one with a *CYP27A1* mutation, two with an *HSD3B7* mutation and one with an *SLCO1B1/SLCO1B3* mutation, and one with an *ABCC2* mutation.

#### Patients With Liver Enzyme Elevation

The diagnostic rate in Group B was 48.6% (34/70), and 25 patients were diagnosed with Wilson disease in this subgroup. A total of 96.0% (24/25) of the patients with Wilson disease harbored compound heterogeneous mutations; only one patient was confirmed as having a homogeneous mutation. Five novel mutations were identified in the patients with Wilson disease. The median age at diagnosis was 46.0 months (IQR: 37.0, 59.5 months). All the patients presented with liver enzyme elevation without other systemic involvement, except one patient who presented with acute liver failure and underwent liver transplantation. Ten patients in this group had one pathogenic heterogeneous mutation, and no pathogenic variant was found among the remaining 27 patients.

Four more patients (5.7%) were diagnosed by NGS, including one patient with an *NBAS* mutation, one with an *MOGS* mutation, one with an IFIH1 mutation and one with an *LAMP2* mutation. Among the five patients suspected of having Wilson disease, three patients had negative results, and two patients had one heterozygous mutation. One patient diagnosed with Wilson disease who developed hepatic cirrhosis was identified as having a homozygous mutation in *ABCB4*.

#### Patients With Hepato/Splenomegaly

The diagnostic rate in this group was 84.6% (33/39), which was the highest among the three groups. GSD was the predominant disease in this group. Twenty-six patients were diagnosed with GSD. Among them, 12 patients had type Ia caused by a *G6PC* mutation, seven patients had type IXa caused by a *PHKA2* mutation, and three had novel mutations. Four patients had type III disease caused by an *AGL* mutation, and one had a novel variant. Three patients had type VI caused by a *PYGL* mutation, and two had novel variants. All GSD patients were under dietary treatment with raw corn starch. None of the patients developed liver failure during follow-up.

One patient diagnosed with GSD was identified as having a homozygous mutation in *GPD1* by NGS. Six patients did not have any disease-related mutations, including three clinically diagnosed GSD patients, one patient with Niemann-Pick disease (NPD), and one patient with Gaucher disease.

### Discussion

Because of the complex clinical manifestations and lack of proper laboratory examinations for some monogenic liver diseases, the incidence of monogenic liver diseases is not known. Nearly half of the chronic liver disorders presenting in childhood have a genetic basis, and ~20% of liver transplantations in children are performed as a consequence of liver-based monogenic disease (7). Most chronic progressive liver diseases except biliary atresia and autoimmune liver disease are inherited liver diseases (8). In different genetic backgrounds, the disease spectrum is completely different. Due to the lack of specific laboratory examinations, most of these diseases could not be precisely diagnosed according to their clinical manifestations before the widespread use of NGS. The disease onset of nearly all inherited liver diseases is within six years of age. A total of 93.8% of the patients diagnosed with monogenic liver disease in this study were enrolled before six years of age. Thus, a proper diagnostic step would help identify monogenic liver disease at an early age.

Currently, the most commonly used genetic testing techniques include TGPS, clinical exome sequencing (CES) and WES. TGPS is focused mainly on a limited number of suspected diseases, and it has better coverage of the genes and is low cost (9). CES includes all genes associated with known diseases, usually including ~4,000 genes (8); here, it is included in the TGPS group. WES is particularly recommended to improve the diagnostic yield in cases of an uncertain phenotype and to discover probable new phenotype-related genes (8). In our cohort, patients with suspected inherited diseases according to initial laboratory tests were studied via TGPS or WES. Otherwise, they were studied via WES. The diagnostic rate between WES and TGPS did not show a significant difference in this study.

#### Patients With Cholestasis

The diagnostic rate in the cholestasis group was nearly 50% in our cohort, which was similar to previously reported data (60%) (10). It was mainly dependent on the criteria to perform sequencing and the sequencing strategy. The disease spectrum in our cohort was different from that in other reports (10). In the study of Nicastro et al. the predominant diagnosis by genetic testing in 50 cholestasis patients was AS, followed by PFIC (six PFIC2 patients, two PFIC3 patents, and one PFIC1 patient). The main population was white people, while in our study, the most common disease found in our cohort was citrin deficiency, which was attributable to the genetic background. Citrin deficiency is common in East Asia and Southeast Asia (11). It is especially common in Northeast China. The most common mutations in our cohort were c.851_854del4 and a 3 kb insertion intron 16. The two pathogenic mutations (c.851_854del and c.1177+1G4A) are mainly seen (30–70%) in the Japanese population (12), while IVS16ins3kb (33%), c.851_854del (30%) and c.1177+1G4A (12%) are the most common pathogenic alleles in the Korean population (13).

AS was the second-ranked disease in Group A in our study. AS is an autosomal dominant disorder with a wide variability in penetrance of clinical features. Approximately 90% of cases are caused by a *JAG1* mutation. However, there is no correlation between genotype and phenotype (14, 15), as not all patients have bile duct paucity (16). In our cohort, seven patients had a *JAG1* mutation, one of whom was diagnosed with AS when he presented with liver dysfunction and portal hypertension. Another six patients had cholestasis, two of them had liver biopsy, and one had bile duct paucity. Notably, the AS patients had unusually high GGT levels. Patient 26 in our cohort with a genetic diagnosis of AS had a normal GGT level. Four mutations were *de novo* mutations, and three of the patients inherited the mutations from their parents, while their parents had no symptoms. All patients of AS with cholestasis were treated with ursodeoxycholic acid, and the jaundice gradually resolved.

Patient 35 was an Alpers syndrome patient. This disease is an inherited disease with both dominant and recessive mutations, which are known to cause a wide spectrum of clinical diseases. Alpers syndrome is caused by mitochondrial DNA depletion and reduced polymerase-γ enzyme activity. It is characterized by intractable seizures, developmental regression, and liver dysfunction (17). Liver dysfunction was reported to occur preceding seizure onset or at the terminal stage of the disease (18). In our cohort, the patient presented with cholestasis, and lactic acidosis preceded the seizure. The patient also had growth failure and developmental retardation. He had intractable epilepticus at the age of six months, and therapy was discontinued.

Patient 31 was considered to have Cerebrotendinous Xanthomatosis (CTX) caused by a *CYP27A1* mutation, which has been associated with infantile cholestasis in several studies (19–21), and it can present as a benign course or severe course. The *CYP27A1* gene encodes CYP27A1 (sterol 27-hydroxylase) (22). The mutation in *CYP27A1* reduces the synthesis of primary bile acids (BA), cholic acid (CA), and chenodeoxycholic acid (CDCA) (23). In our case, the patient was treated with UDCA and had a benign clinical course.

#### Patients With Liver Enzyme Elevation

The predominant diagnosis in this group was Wilson disease. Most patients diagnosed with Wilson disease in this group were screened out by liver function tests when having a healthy examination at the time of admission to kindergarten or by accident. Wilson disease could be established by serum ceruloplasmin levels and 24-h urine copper levels. The serum ceruloplasmin level was dramatically decreased in all the patients in this cohort. Monogenic liver disease often presents with acute liver failure as well. Biallelic mutations in the *NBAS* gene have been identified in recurrent acute liver failure in early infancy. The clinical spectrum of patients with dysfunctions in the gene *NBAS* is variable. Some patients had a multisystemic disease with short stature, skeletal dysplasia, immunological abnormalities, optic atrophy, and normal motor and cognitive development resembling SOPH syndrome (24). The patient with the NBAS mutation in this study had liver failure that was triggered by fever, which was then accompanied by seizure and consciousness problems. We did not observe any other accompanying symptoms. Another two patients with acute liver failure had a heterozygous *NBAS* mutation in this study.

Congenital disorders of glycosylation (CDG) type IIb is an extremely rare CDG that has only been reported in six patients to the best of our knowledge. CDG type IIb has multisystemic abnormalities and distinct dysmorphic features (25). The patient in this study presented developmental retardation and liver dysfunction. His brother had similar symptoms and died, while his elder sister was normal. He had two novel compound mutations in *MOGS* that have not been reported.

#### Patients With Hepatomegaly or Hepatosplenomegaly

Patients presenting with hepatomegaly or hepatosplenomegaly have a high susceptibility to metabolic liver diseases. The diagnostic rate of monogenic liver disease in this cohort was 84.6%, the majority of cases were GSD. There was a high consistency between the genetic diagnosis and clinical diagnosis or histology of liver biopsy. Clinical and laboratory findings, including hepatomegaly, hypoglycemia, hyperlacticaemia, hyperlipidemia, and growth retardation, are useful in the diagnosis of GSD. However, genetic tests can precisely identify the form of GSD according to the gene mutation. Very long-chain acyl-CoA dehydrogenase deficiency (VLCADD) is a rare metabolic disorder of mitochondrial fatty acid oxidation. The clinical manifestation of VLCADD is variable and is divided into three categories according to severity: the severe early-onset cardiac and multiorgan failure form, the hepatic or hypoketotic hypoglycemic form, and the later-onset episodic myopathic form (26). Our patient presented with mild clinical manifestations of hepatosplenomegaly, liver dysfunction and elevation of anomia. Biallelic mutations in the *GPD1* gene cause a rare autosomal recessive inherited disease known as transient infantile hypertriglyceridemia. To date, fewer than 20 patients have been reported. Only one adolescent patient presented obesity, insulin resistance, fatty liver, and short stature (27). Our patient was misdiagnosed with GSD according to the clinical findings. The histological study of the liver biopsy in another hospital indicated fatty liver. Genetic sequencing found a novel mutation of *GPD1*, and it is the only homogeneous mutation of *GPD1* reported to date.

The use of NGS in the diagnosis of inherited liver disease has a high diagnostic rate. The duration to diagnosis is shortened to two weeks to one month, and the cost of NGS is dramatically reduced. Most patients could obtain genetic diagnosis reports within 3 weeks. Parents of patients accept NGS more easily than other conventional diagnostic methods, such as liver biopsy. Twelve and four more patients were diagnosed by NGS in Group A and Group B, respectively. Five patients in group C were misdiagnosed with genetic liver disease and were mainly misdiagnosed with GSD by initial laboratory workup. One rare disease caused by a *GPD1* mutation was diagnosed after NGS testing. Early diagnosis of these patients could lead to more precise treatment. NGS would help classify the type of GSD patients as well. The main limitation of this study is that the diagnosis of these monogenic liver diseases was based only on clinical, laboratory and genetic findings. To obtain a definitive diagnosis of some diseases, a functional study is also required. The second limitation is that the patients were only recruited from the gastroenterology department of one tertiary hospital. The disease spectrum here did not include some severe monogenic liver diseases that had disease onset during the neonatal period and were diagnosed during the neonatal period or passed without definite diagnosis.

In conclusion, a large number of pediatric liver diseases are caused by genetic disorders. NGS showed a high diagnosis rate in patients presenting with various clinical manifestations. Both WES and TGPS had similar diagnostic rates, while TGPS was more economical. Patients with other involved systems or with obvious hepatosplenomegaly had the highest rate of genetic disorders in this cohort.

## Data Availability Statement

The data used to support the findings of this study are included within the article. The sequencing data of all the patients is available on the servers of the gene sequencing companies (MyGenostics Inc. and RunningGene Inc.).

## Ethics Statement

The studies involving human participants were reviewed and approved by The Children's Hospital, Zhejiang University School of Medicine. Written informed consent to participate in this study was provided by the participants' legal guardian/next of kin.

## Author Contributions

YF and JC designed the research. YF drafted the manuscript. JY, KP, and HZ collected the patients information. All authors contributed to the article and approved the submitted version.

## Conflict of Interest

The authors declare that the research was conducted in the absence of any commercial or financial relationships that could be construed as a potential conflict of interest.

## References

[1] Stalke A Skawran B Auber B Illig T Schlegelberger B Junge N. Diagnosis of monogenic liver diseases in childhood by next-generation sequencing. Clin Genet. (2018) 93:665–70. 10.1111/cge.13120 28776642

[2] Fawaz R Baumann U Ekong U Fischler B Hadzic N Mack CL. Guideline for the evaluation of cholestatic jaundice in infants: Joint Recommendations of the North American Society for Pediatric Gastroenterology, Hepatology, and Nutrition and the European Society for Pediatric Gastroenterology, Hepatology, and Nutrition. J Pediatr Gastroenterol Nutr. (2017) 64:154–68. 10.1097/MPG.0000000000001334 27429428

[3] Fang YH Luo YY Yu JD Lou JG Chen J. Phenotypic and genotypic characterization of inflammatory bowel disease in children under six years of age in China. World J Gastroenterol. (2018) 24:1035–45. 10.3748/wjg.v24.i9.1035 29531467 PMC5840468

[4] Richards S Aziz N Bale S Bick D Das S Gastier-Foster J. Standards and guidelines for the interpretation of sequence variants: a joint consensus recommendation of the American College of Medical Genetics and Genomics and the Association for Molecular Pathology. Genet Med. (2015) 17:405–24. 10.1038/gim.2015.30 25741868 PMC4544753

[5] Song YZ Deng M Chen FP Wen F Guo L Cao SL. Genotypic and phenotypic features of citrin deficiency: five-year experience in a Chinese pediatric center. Int J Mol Med. (2011) 28:33–40. 10.3892/ijmm.2011.653 21424115

[6] Chong SC Lo P Chow CW Yuen L Chu WCW Leung TY. Molecular and clinical characterization of citrin deficiency in a cohort of Chinese patients in Hong Kong. Mol Genet Metab Rep. (2018) 17:3–8. 10.1016/j.ymgmr.2018.08.002 30181955 PMC6120422

[7] Sze YK Dhawan A Taylor RM Bansal S Mieli-Vergani G Rela M. Pediatric liver transplantation for metabolic liver disease: experience at King's College Hospital. Transplantation. (2009) 87:87–93. 10.1097/TP.0b013e31818bc0c4 19136896

[8] Nicastro E D'Antiga L. Next generation sequencing in pediatric hepatology and liver transplantation. Liver Transpl. (2018) 24:282–93. 10.1002/lt.24964 29080241

[9] Kingsmore SF Saunders CJ. Deep sequencing of patient genomes for disease diagnosis: when will it become routine? Sci Transl Med. (2011) 3:87ps23. 10.1126/scitranslmed.3002695 21677196 PMC4264992

[10] Nicastro E Di Giorgio A Marchetti D Barboni C Cereda A Iascone M. Diagnostic yield of an algorithm for neonatal and infantile cholestasis integrating next-generation sequencing. J Pediatr. (2019) 211:54–62. 10.1016/j.jpeds.2019.04.016 31160058

[11] Okano Y Ohura T Sakamoto O Inui A. Current treatment for citrin deficiency during NICCD and adaptation/compensation stages: strategy to prevent CTLN2. Mol Genet Metab. (2019) 127:175–83. 10.1016/j.ymgme.2019.06.004 31255436

[12] Tabata A Sheng JS Ushikai M Song YZ Gao HZ Lu YB. Identification of 13 novel mutations including a retrotransposal insertion in SLC25A13 gene and frequency of 30 mutations found in patients with citrin deficiency. J Hum Genet. (2008) 53:534–45. 10.1007/s10038-008-0282-2 18392553

[13] Oh SH Lee BH Kim GH Choi JH Kim KM Yoo HW. Biochemical and molecular characteristics of citrin deficiency in Korean children. J Hum Genet. (2017) 62:305–7. 10.1038/jhg.2016.131 27829683

[14] Kamath BM Bauer RC Loomes KM Chao G Gerfen J Hutchinson A. NOTCH2 mutations in Alagille syndrome. J Med Genet. (2012) 49:138–44. 10.1136/jmedgenet-2011-100544 22209762 PMC3682659

[15] Mitchell E Gilbert M Loomes KM. Alagille Syndrome. Clin Liver Dis. (2018) 22:625–41. 10.1016/j.cld.2018.06.001 30266153

[16] Subramaniam P Knisely A Portmann B Qureshi SA Aclimandos WA Karani JB. Diagnosis of Alagille syndrome-25 years of experience at King's College Hospital. J Pediatr Gastroenterol Nutr. (2011) 52:84–9. 10.1097/MPG.0b013e3181f1572d 21119543

[17] Saneto RP Cohen BH Copeland WC Naviaux RK. Alpers-Huttenlocher syndrome. Pediatr Neurol. (2013) 48:167–78. 10.1016/j.pediatrneurol.2012.09.014 23419467 PMC3578656

[18] Egger J Harding BN Boyd SG Wilson J Erdohazi M. Progressive neuronal degeneration of childhood (PNDC) with liver disease. Clin Pediatr (Phila). (1987) 26:167–73. 10.1177/000992288702600401 2435443

[19] Clayton PT Verrips A Sistermans E Mann A Mieli-Vergani G Wevers R. Mutations in the sterol 27-hydroxylase gene (CYP27A) cause hepatitis of infancy as well as cerebrotendinous xanthomatosis. J Inherit Metab Dis. (2002) 25:501–13. 10.1023/A:1021211520034 12555943

[20] Huidekoper HH Vaz FM Verrips A Bosch AM. Hepatotoxicity due to chenodeoxycholic acid supplementation in an infant with cerebrotendinous xanthomatosis: implications for treatment. Eur J Pediatr. (2016) 175:143–6. 10.1007/s00431-015-2584-7 26156051 PMC4709371

[21] Gong JY Setchell KDR Zhao J Zhang W Wolfe B Lu Y. Severe Neonatal cholestasis in Cerebrotendinous Xanthomatosis: genetics, immunostaining, mass spectrometry. J Pediatr Gastroenterol Nutr. (2017) 65:561–8. 10.1097/MPG.0000000000001730 28937538

[22] Oftebro H Bjorkhem I Skrede S Schreiner A Pederson JI. Cerebrotendinous xanthomatosis: a defect in mitochondrial 26-hydroxylation required for normal biosynthesis of cholic acid. J Clin Invest. (1980) 65:1418–30. 10.1172/JCI109806 7410549 PMC371480

[23] Setoguchi T Salen G Tint GS Mosbach EH. A biochemical abnormality in cerebrotendinous xanthomatosis. Impairment of bile acid biosynthesis associated with incomplete degradation of the cholesterol side chain. J Clin Invest. (1974) 53:1393–401. 10.1172/JCI107688 4825231 PMC302628

[24] Staufner C Haack TB Kopke MG Straub BK Kolker S Thiel C. Recurrent acute liver failure due to NBAS deficiency: phenotypic spectrum, disease mechanisms, and therapeutic concepts. J Inherit Metab Dis. (2016) 39:3–16. 10.1007/s10545-015-9896-7 26541327

[25] Kim YM Seo GH Jung E Jang JH Kim SZ Lee BH. Characteristic dysmorphic features in congenital disorders of glycosylation type IIb. J Hum Genet. (2018) 63:383–6. 10.1038/s10038-017-0386-7 29235540

[26] Yamada K Taketani T. Management and diagnosis of mitochondrial fatty acid oxidation disorders: focus on very-long-chain acyl-CoA dehydrogenase deficiency. J Hum Genet. (2019) 64:73–85. 10.1038/s10038-018-0527-7 30401918

[27] Li N Chang G Xu Y Ding Y Li G Yu T. Biallelic mutations in GPD1 gene in a Chinese boy mainly presented with obesity, insulin resistance, fatty liver, and short stature. Am J Med Genet A. (2017) 173:3189–94. 10.1002/ajmg.a.38473 28944580

