# Supplementary material for: Clinical and Genetic Spectra of Inherited Liver Disease in Children in China
Source: Front Pediatr. 2021 Mar 4;9:631620. doi: 10.3389/fped.2021.631620 (PMC7982861; doi:10.3389/fped.2021.631620)
Supplement: Supplementary file 1 [file Data_Sheet_1.PDF]

Supplemnet table 1. The geography information and results of next generation sequencing of 172 patients.

Number	Gender	Group	Family history	Clinical diagnosis	Age (M)	WES/ TGPS	Mutation	Genetic diagnosis	Gene	Pathogenic variants	Location of mutation	Type of mutation	Inheritance	New variant	SIFT score/ prediction	Polyphen2 score/ prediction	MutationTaster score/ prediction	Pathogenic
1	F	A	NO	Citrin defiency	2.6	TGPS	Com	Citrin defiency	SLC25A13	_	C.852_855delTATG<br>chr7:95818684<br>P.Met285Profs*2<br>IVS16ins3Kb	deletion mutation<br>insertion mutation	Mother<br>Father	NO<br>NO	_	_	_	P<br>P
2	F	A	NO	Citrin defiency	2.8	TGPS	Hom	Citrin defiency	SLC25A13	_	C.852_855delTATG<br>chr7:95818684<br>P.Met285Profs*2	deletion mutation	Mother<br>Father	NO	_	_	_	p
3	F	A	NO	Citrin defiency	2.6	TGPS	Hom	Citrin defiency	SLC25A13	_	IVS16ins3Kb	insertion mutation	Mother<br>Father	NO	_	_	_	p
4	F	A	NO	Citrin defiency	2.5	TGPS	Het	Negative	SLC25A13	YES	c.615+5G>A<br>chr7:95822344<br>splicing	splicing mutation	Mother	NO	_	_	_	P
5	F	A	NO	Citrin defiency	2.3	TGPS	Com	Citrin defiency	SLC25A13	_	c.1402C>T<br>chr7:95775921<br>p.R468*<br>c.475C>T<br>chr7:95822489<br>p.Q159*	nonsense mutation<br>nonsense mutation	Mother<br>Father	NO<br>YES	_<br>_	_<br>_	Damaging<br>Damaging	P<br>P
6	F	A	NO	Citrin defiency	5.3	TGPS	Com	Citrin defiency	SLC25A13	_	C.852_855delTATG<br>chr7:95818684<br>P.Met285Profs*2<br>IVS16ins3Kb	deletion mutation<br>insertion muation	Father<br>Mother	NO<br>NO	_<br>_	_<br>_	_<br>_	P<br>P
7	M	A	NO	Citrin defiency	5.1	TGPS	Hom	Citrin defiency	SLC25A13	_	C.852_855delTATG<br>chr7:95818684<br>P.Met285Profs*2	deletion mutation	Mother<br>Father	NO	_	_	_	P
8	M	A	NO	Citrin defiency	1.9	TGPS	Com	Citrin defiency	SLC25A13	_	C.852_855delTATG<br>chr7:95818684<br>P.Met285Profs*2<br>c.401T>A<br>chr7:95838217<br>p.V134E	deletion mutation<br>missense mutation	Mother<br>Father	NO<br>YES	_<br>Damaging	_<br>Possibility damaging	_<br>damaging	p<br>LP
9	M	A	NO	Citrin defiency	1.8	TGPS	Com	Citrin defiency	SLC25A13	_	c.1078C>T<br>chr7:95813688<br>p.R360*<br>C.852_855delTATG<br>chr7:95818684<br>P.Met285Profs*2	nonsense mutation<br>deletion mutation	Father<br>Mother	NO<br>NO	_<br>_	_<br>_	Damaging<br>_	p<br>P
10	M	A	NO	Citrin defiency	1.2	TGPS	Hom	Citrin defiency	SLC25A13	_	C.852_855delTATG<br>chr7:95818684<br>P.Met285Profs*2	deletion mutation	Mother<br>Father	NO	_	_	_	P
11	M	A	NO	Cholestatic hepatitis	1.7	WES	Hom	Citrin defiency	SLC25A13	_	C.852_855delTATG<br>chr7:95818684<br>P.Met285Profs*2	deletion mutation	Mother<br>Father	NO	_	_	_	P
12	F	A	NO	Citrin defiency	5.2	TGPS	Hom	Citrin defiency	SLC25A13	_	C.852_855delTATG<br>chr7:95818684<br>P.Met285Profs*2	deletion mutation	Mother<br>Father	NO	_	_	_	P
13	F	A	NO	Citrin defiency	5.0	WES	Hom	Citrin defiency	SLC25A13	_	C.852_855delTATG<br>chr7:95818684<br>P.Met285Profs*2	deletion mutation	Mother<br>Father (not available）	NO	_	_	_	P
14	F	A	NO	Citrin defiency	1.7	WES	Com	Citrin defiency	SLC25A13	_	C.852_855delTATG<br>chr7:95818684<br>P.Met285Profs*2<br>c.615+5G>A<br>chr7-95822344*2<br>splice	deletion mutation<br>splicing mutation	Father<br>Mother	NO<br>NO	_<br>_	_<br>_	_<br>_	P<br>p
15	M	A	NO	Citrin defiency	2.5	WES	Com	Citrin defiency	SLC25A13	_	C.852_855delTATG<br>chr7:95818684<br>P.Met285Profs*2<br>c.1813C>T<br>chr7-95750995<br>p.R605*	deletion mutation<br>nonsense mutation	Mother<br>Father	NO<br>NO	_<br>_	_<br>_	_<br>Damaging	P<br>P
16	F	A	NO	Citrin defiency	2.0	TGPS	Com	Citrin defiency	SLC25A13	_	c.289C>T<br>chr7:95864153<br>p.Q97*<br>Intron 6 c.615+5G>A<br>chr7:95822344<br>splice	nonsense mutation<br>splicing mutation	Mother<br>Father	YES<br>NO	_<br>_	_<br>_	Damaging<br>_	P<br>P
17	F	A	NO	Citrin defiency	2.5	TGPS	Com	Citrin defiency	SLC25A13	_	c.1078C>T<br>chr7:95813688<br>p.R360*<br>c.754G>A<br>chr7:95820421<br>p.E252K	nonsense mutation<br>missense mutation	Mother<br>Father	NO<br>NO	_<br>damaging	_<br>Possibility damaging	Damaging<br>Damaging	p<br>LP
18	F	A	NO	PFIC	12.0	TGPS	Het	Negative	TJP2	YES	c.1010C>G<br>chr9:71840265<br>p.T337S	missense mutation	Mother	YES	Tolerated	Probably damaging	Damaging	VUS
19	M	A	NO	PFIC	8.0	TGPS	Het	Negative	ABCB11	YES	c.3458G>A<br>chr2:169783826<br>p.R1153H	missense mutation	Father	NO	Damaging	Probably damaging	Damaging	LP

20	F	A	NO	PFIC	2.1	WES	Com	PFIC 2	ABCB11	_	c.667C>T<br>chr2:169850337<br>p.R223C<br>intron14:c.1639-4C>G<br>chr2:169826729<br>splice	missense mutation<br>splicing mutation	Father<br>Mother	NO<br>YES	Tolerated<br>_	Probably damaging<br>_	Damaging<br>_	VUS<br>VUS
21	F	A	NO	Cholestatic hepatitis	2.2	TGPS	Het	Alagille syndrome	JAG1	_	c.887-2A>G<br>chr20:10632900<br>splicing	splicing mutation	_	YES	_	_	Damaging	LP
22	M	A	NO	Cholestatic hepatitis	6.4	TGPS	Het	Alagille syndrome	JAG1	_	c.694+1G>A<br>chr20:10639115<br>splicing	splicing mutation	Mother	YES	_	_	Damaging	LP
23	M	A	NO	Alagille syndrome	48.0	TGPS	Het	Alagille syndrome	JAG1	_	c.487C>A<br>chr20:10639323<br>p.P163T	missense mutation	_	YES	Damaging	Probably damaging	Damaging	VUS
24	M	A	NO	Cholestatic hepatitis	4.7	TGPS	Het	Alagille syndrome	JAG1	_	c.270delG<br>chr20:10653466<br>p.Cys92Alafs*69	deletion mutation	_	NO	_	_	_	P
25	M	A	NO	Cholestatic hepatitis	68.0	WES	Het	Alagille syndrome	JAG1	_	c.2850delT<br>chr20:10622174<br>p.Asp951Thrfs*19	deletion mutation	_	YES	_	_	_	P
26	M	A	NO	Cholestatic hepatitis	2.5	WES	Het	Alagille syndrome	JAG1	_	c.550C>G<br>chr20:10639260<br>p.R184G	missense mutation	Mother	NO	Damaging	Probably damaging	Damaging	VUS
27	M	A	NO	Citrin defiency	5.4	TGPS	Het	Negative	SLC25A13	YES	IVS16ins3Kb	insertion mutation	denovo	NO	–	–	–	P
28	M	A	NO		18.0	TGPS	Neg	Negative	_	_	_	_	_	_	_	_	_	_
29	F	A	NO	Citrin defiency	3.0	TGPS	Neg	Negative	_	_	_	_	_	_	_	_	_	_
30	F	A	NO	Citrin defiency	9.0	TGPS	Neg	Negative	_	_	_	_	_	_	_	_	_	_
31	M	A	NO	Cholestatic hepatitis	4.0	TGPS	Hom	CTX	CYP27A1	_	c.1420C>T<br>chr2:219679424<br>p.R474W	missense mutation	Mother<br>Father	NO	Damaging	Probably damaging	Damaging	p
32	M	A	NO	Cholestatic hepatitis	1.5	TGPS	Neg	Negative	_	_	_	_	_	_	_	_	_	_
33	F	A	NO	Cholestatic hepatitis	24.0	TGPS	Neg	Negative	_	_	_	_	_	_	_	_	_	_
34	F	A	NO	Cholestatic hepatitis	3.4	WES	Neg	Negative	_	_	_	_	_	_	_	_	_	_
35	M	A	NO	Cholestatic hepatitis	5.0	TGPS	Com	Alpers syndrome	POLG	_	c.3403G>C<br>chr15:89861851<br>p.D1135H<br>c.2890C>T<br>chr15:89864088<br>p.R964C	missense mutation<br>missense mutation	Father<br>Mother	YES<br>NO	Damaging<br>Damaging	Probably damaging<br>Possibly damaging	Damaging<br>Damaging	VUS<br>P
36	M	A	NO	Cholestatic hepatitis	2.3	TGPS	Neg	Negative	_	_	_	_	_	_	_	_	_	_
37	F	A	NO	Cholestatic hepatitis	2.6	TGPS	Neg	Negative	_	_	_	_	_	_	_	_	_	_
38	F	A	NO	Genetic liver diseas	120.0	TGPS	Het	Negative	CPT2	YES	c.4G>A<br>chr1:53662619<br>p.V2M	missense mutation	Mother	YES	Tolerated	Benign	Tolerated	VUS
39	M	A	NO	Acute liver failure	108.0	TGPS	Neg	Negative	_	_	_	_	_	_	_	_	_	_
40	M	A	NO	Cholestatic hepatitis	2.3	WES	Neg	Negative	_	_	_	_	_	_	_	_	_	_

41	M	A	NO	Cholestatic hepatitis	2.0	WES	Com	IEBAS	HSD3B7	_	c.503G>A<br>chr16:30997997<br>p.W168*<br>c.743G>C<br>chr16:30999137<br>p.R248P	nonsense mutation<br>missense mutation	Mother<br>de novo	NO<br>YES	_<br>Tolerated	_<br>Benign	Damaging<br>Tolerated	p<br>LP
42	F	A	NO	Cholestatic hepatitis	2.5	WES	Het	Negative	TREX1	YES	c.144dupC<br>chr3-48508191<br>p.T49Hfs*53	insertion mutation	Father	NO	_	_	_	p
43	F	A	NO	Cholestatic hepatitis	144.0	WES	Neg	Negative	_	_	_	_	_	_	_	_	_	_
44	F	A	NO	PFIC	8.1	WES	Neg	Negative	_	_	_	_	_	_	_	_	_	_
45	M	A	NO	Acute liver failure	5.8	WES	Neg	Negative	_	_	_	_	_	_	_	_	_	_
46	F	A	NO	Cholestatic hepatitis	2.4	WES	Neg	Negative	_	_	_	_	_	_	_	_	_	_
47	M	A	NO	Cholestatic hepatitis	2.2	TGPS	Neg	Negative	_	_	_	_	_	_	_	_	_	_
48	M	A	NO	Cholestatic hepatitis	2.4	TGPS	Neg	Negative	_	_	_	_	_	_	_	_	_	_
49	F	A	NO	Algille syndrome	10.0	WES	Neg	Negative	_	_	_	_	_	_	_	_	_	_
50	F	A	NO	Cholestatic hepatitis	56.0	WES	Hom	Roter syndrome	SLCO1B1/<br>SLCO1B3	_	c.1738C>T<br>chr12-21375289<br>p.R580*<br>intron	nonsense mutation<br>deletion	Mother Father<br>Mother Father	NO<br>_	_<br>_	_<br>_	Damaging<br>_	p<br>VUS
51	M	A	NO	Cholestatic hepatitis	7.2	WES	Com	IEBAS	HSD3B7<br><br>SLC25A13	_<br>_<br>YES	c.402_403insG<br>chr16:30997819<br>p.P135Afs*2<br>c.503G>A<br>chr16-30997997<br>p.W168*<br>c.2T>C<br>chr7:95951267<br>p.M1*	insertion mutation<br>nonsense mutation<br>nonsense mutation	Mother<br>Father<br>Father	YES<br>NO<br>NO	_<br>_<br>Damaging	_<br>_<br>Probabaly damaging	_<br>Damaging<br>Damaging	P<br>P<br>VUS
52	M	A	NO	Genetic liver diseas	2.0	WES	Het	Negative	OTC	YES	_	_	Not vavidate	_	_	_	_	_
53	M	A	NO	Cholestatic hepatitis	92.0	WES	Het	Negative	HSD3B7	YES	c.521A>G<br>chr16:30998015<br>p.N174S	missense mutation	Father	YES	Damaging	Probably damaging	Damaging	VUS
54	M	A	NO	Cholestatic hepatitis	123.0	WES	Neg	Negative	_	_	_	_	_	_	_	_	_	_
55	M	A	NO	Acute liver failure	10.0	WES	Neg	Negative	_	_	_	_	_	_	_	_	_	_
56	M	A	NO	Acute liver failure	2.9	WES	Het	Negative	_	_	_	_	_	_	_	_	_	_
57	M	A	NO	Cholestatic hepatitis	10.0	TGPS	Neg	Negative	_	_	_	_	_	_	_	_	_	_
58	M	A	NO	Citrin defiency	4.9	TGPS	Neg	Negative	_	_	_	_	_	_	_	_	_	_
59	F	A	NO	Cholestatic hepatitis	104.0	WES	Neg	Negative	_	_	_	_	_	_	_	_	_	_
60	M	A	NO	Cholestatic hepatitis	14.0	WES	Het	Negative	ABCB4	YES	c.1175G>A<br>chr7:87073034<br>p.G392E	missense mutation	Mother	YES	Damaging	Probably damaging	Damaging	VUS
61	M	A	NO	Cholestatic hepatitis	13.0	TGPS	Hom	Dubin-Johnson syndrome	ABCC2	_	c.1586G>A<br>chr10:101567196<br>p.R529Q	missense mutation	Mother Father	YES	Damaging	Probably damaging	Damaging	VUS

62	F	A	NO	Cholestatic hepatitis	96.0	WES	Het	Negative	CPOX	YES	c.1339C>T chr3:98299553 p.R447C	missense mutation	Mother	NO	Damaging	Probably damaging	Damaging	VUS
63	M	A	NO	Cholestatic hepatitis	2.0	TGPS	Het	Negative	MAT1A	YES	c.227T>C chr10:82043737 p.M76T	missense mutation	Father	_	_	_	_	VUS
64	M	B	NO	Wilson's disease	48.0	TGPS	Com	Wilson's disease	ATP7B	_	c.2755C>G chr13:52523908 p.R919G<br>c.2333G>T chr13:52532469 p.R778L<br>c.2310C>G chr13:52532492 p.L770L	missense mutation<br>missense mutation<br>synonymous mutation	Mother<br>Father<br>Father	NO<br>NO<br>NO	Damaging<br>Damaging<br>_	Probably damaging<br>Possibly damaging<br>_	Damaging<br>Damaging<br>–	P<br>P<br>VUS
65	M	B	NO	Wilson's disease	24.0	TGPS	Com	Wilson's disease	ATP7B	_	c.2333G>T chr13:52532469 p.R778L<br>c.2292C>T chr13:52532510 p.F764F	missense mutation<br>missense mutation	Mother<br>Father	NO<br>YES	Damaging<br>_	Probably damaging<br>_	Damaging<br>_	P<br>VUS
66	M	B	NO	Wilson's disease	36.0	TGPS	Com	Wilson's disease	ATP7B	_	c.2135C>T chr13:52524252 p.A712V<br>c.994G>T chr13:52548362 p.E332*	missense mutation<br>nonsense mutation	Father<br>Mother	NO<br>NO	Damaging<br>_	Probably damaging<br>_	Damaging<br>Damaging	P<br>P
67	F	B	NO	Wilson's disease	60.0	TGPS	Com	Wilson's disease	ATP7B	_	c.2336C>T chr13:52520523 p.S779F<br>c.2333G>T chr13:52532469 p.R778L	missense mutation<br>missense mutation	Mother<br>Father	NO<br>NO	Damaging<br>Damaging	Probably damaging<br>Possibly damaging	Damaging<br>Damaging	P<br>P
68	F	B	NO	Wilson's disease	72.0	TGPS	Com	Wilson's disease	ATP7B	_	c.2333G>T chr13:52532469 p.R778L<br>c.1708-1G>C chr13:52539170 splicing<br>c.1168A>G chr13:52548188 p.I390V	missense mutation<br>splicing mutation<br>missense mutation	Mother<br>Father<br>Father	NO<br>YES<br>NO	Damaging<br>_<br>Tolerate	Probably damaging<br>_<br>Benign	Damaging<br>Damaging<br>Tolerate	P<br>P<br>VUS
69	F	B	NO	Wilson's disease	41.0	TGPS	Com	Wilson's disease	ATP7B	_	c.3443T>C chr13:52515330 p.I1148T<br>c.2975C>T chr13:52520505 p.P992L	missense mutation<br>missense mutation	Father<br>Mother	NO<br>NO	Damaging<br>Damaging	Probably damaging<br>Possibly damaging	Damaging<br>Damaging	P<br>P
70	F	B	NO	Wilson's disease	25.0	TGPS	Com	Wilson's disease	ATP7B	_	c.2804C>T chr13:52523859 p.T935M<br>c.2333G>T chr13:52532469 p.R778L<br>c.2310C>G chr13:52532492 p.L770L	missense mutation<br>missense mutation<br>synonymous mutation	Father<br>Mother<br>Mother	NO<br>NO<br>NO	Damaging<br>Damaging<br>_	Probably damaging<br>Possibly damaging<br>_	Damaging<br>Damaging<br>_	P<br>P<br>VUS
71	F	B	NO	Wilson's disease	72.0	TGPS	Com	Wilson's disease	ATP7B	_	c.3008C>T chr13:52520472 p.A1003V<br>c.2333G>T chr13:52532469 p.R778L<br>c.2310C>G chr13:52532492 p.L770L	mutation<br>missense mutation<br>synonymous mutation	Father<br>Mother<br>Mother	NO<br>NO<br>NO	Damaging<br>Damaging<br>_	Probably damaging<br>Possibly damaging<br>_	Damaging<br>Damaging<br>_	P<br>P<br>VUS
72	F	B	NO	Wilson's disease	36.0	TGPS	Com	Wilson's disease	ATP7B	_	c.2975C>T chr13:52520505 p.P992L<br>c.2333G>T chr13:52532469 p.R778L<br>c.2310C>G chr13:52532492 p.L770L	missense mutation<br>missense mutation<br>synonymous mutation	Father<br>Mother<br>Mother	NO<br>NO<br>NO	Damaging<br>Damaging<br>_	Probably damaging<br>Possibly damaging<br>_	Damaging<br>Damaging<br>_	P<br>P<br>VUS
73	M	B	NO	Wilson's disease	44.0	TGPS	Com	Wilson's disease	ATP7B	_	c.3700delG chr13:52511815 p.V1234fs*96<br>c.2333G>T chr13:52532469 p.R778L	deletion mutation<br>missense mutation	Father<br>Mother	NO<br>NO	_<br>Damaging	_<br>Possibly damaging	_<br>Damaging	P<br>P
74	M	B	NO	Wilson's disease	41.0	TGPS	Com	Wilson's disease	ATP7B	_	c.3562C>T chr13:52513324 p.L1188F<br>c.2975C>T chr13:52520505 p.P992L	missense mutation<br>missense mutation	Mother<br>Father	YES<br>NO	Damaging<br>Damaging	Probably damaging<br>Possibly damaging	Damaging<br>Damaging	VUS<br>P
75	M	B	NO	Wilson's disease	49.0	TGPS	Com	Wilson's disease	ATP7B	_	c.3517G>A chr13-52515256*1 p.E1173K<br>c.2333G>T chr13-52532469*2 p.R778L<br>c.2310C>G chr13-52532492*3 p.L770L	missense mutation<br>missense mutation<br>synonymous mutation	Father<br>Mother<br>Mother	NO<br>NO<br>NO	Damaging<br>Damaging<br>_	Probably damaging<br>Possibly damaging<br>_	Damaging<br>Damaging<br>_	P<br>P<br>VUS
76	F	B	NO	Wilson's disease	59.0	TGPS	Com	Wilson's disease	ATP7B	_	c.2975C>T chr13-52520505 p.P992L<br>c.2297C>T chr13-52532505*2 p.T766M	missense mutation<br>missense mutation	Father<br>Mother	NO<br>NO	Damaging<br>Damaging	Probably damaging<br>Possibly damaging	Damaging<br>Damaging	P<br>P
77	F	B	NO	Wilson's disease	50.0	TGPS	Com	Wilson's disease	ATP7B	_	c.1586A>G chr13-52542701 p.E529G<br>c.367delG chr13-52548989*2 p.A123Pfs*30	missense mutation<br>deletion mutation	Father<br>Mother	NO<br>NO	_<br>_	_<br>_	_<br>_	P<br>P

78	M	B	NO	Wilson's disease	40.0	WES	Com	Wilson's disease	ATP7B	_	c.525dupA chr13-52548830 p.V176Sfs*28<br>c.2804C>T chr13-52523859 p.T935M	insertion mutation<br>missense mutation	Father<br>Mother	NO<br>NO	_<br>Damaging	_<br>Possibly damaging	_<br>Damaging	P<br>P
79	F	B	NO	Wilson's disease	39.0	TGPS	Hom	Wilson's disease	ATP7B	_	c.2333G>T chr13:52532469 p.R778L	missense mutation	Mother<br>Father	NO	Damaging	Probably damaging	Damaging	LP
80	M	B	NO	Wilson's disease	46.0	TGPS	Com	Wilson's disease	ATP7B	_	c.2975C>T chr13:52520505 p.P992L<br>chr13:52523908 c.2755C>G p.R919G	missense mutation<br>missense mutation	Mother<br>Father	NO<br>NO	Damaging<br>Damaging	Probably damaging<br>Possibly damaging	Damaging<br>Damaging	P<br>P
81	F	B	NO	Wilson's disease	51.0	TGPS	Com	Wilson's disease	ATP7B	_	c.2333G>T chr13:52532469 p.R778L<br>chr13:52542716 c.1571T>C p.M524T	missense mutation<br>missense mutation	Father<br>Mother	NO<br>YES	Damaging<br>Damaging	Probably damaging<br>Possibly damaging	Damaging<br>Damaging	P<br>LP
82	M	B	NO	Wilson's disease	83.0	TGPS	Com	Wilson's disease	ATP7B	_	c.2333G>T chr13:52532469 p.R778L<br>chr13:52523908 c.2755C>G p.R919G	missense mutation<br>missense mutation	Mother<br>Father	NO<br>NO	Damaging<br>Damaging	Probably damaging<br>Possibly damaging	Damaging<br>Damaging	P<br>P
83	F	B	NO	Wilson's disease	24.0	TGPS	Com	Wilson's disease	ATP7B	_	c.2333G>T chr13-52532469 p.R778L<br>c.2804C>T chr13-52523859 p.T935M<br>c.2310C>G chr13-52532492 p.L770L	missense mutation<br>missense mutation<br>synonymou s mutation	Father<br>Mother<br>Father	NO<br>NO<br>NO	Damaging<br>Damaging<br>_	Probably damaging<br>Possibly damaging<br>_	Damaging<br>Damaging<br>_	P<br>P<br>VUS
84	F	B	NO	Wilson's disease	48.0	TGPS	Com	Wilson's disease	ATP7B	_	c.2304_2305insC chr13-52532497 p.M769Hfs*26<br>c.2621C>T chr13-52524252 p.A874V	insertion mutation<br>missense mutation	Father<br>Mother	NO<br>NO	_<br>Damaging	_<br>Possibly damaging	_<br>Damaging	P<br>P
85	M	B	NO	Algille syndrome?	51.0	WES	Het	Alagille syndrome	JAG1	_	c.35_45delGCCCCCT AAGC chr20:10654134-10654144 p.R12Pfs*57	deletion mutation	Father	YES	_	_	_	LP
86	F	B	NO	Liver dysfunciton	4.5	TGPS	Het	Negative	NBAS	YES	c.5162A>G chr2:15417202 p.N1721S<br>c.2572C>T chr2:15564444 p.R858W	missense mutation<br>missense mutation	Father<br>Father	YES<br>YES	Tolerated<br>Damaging	Benign<br>Possibly damaging	Tolerated<br>Tolerated	VUS<br>VUS
87	M	B	NO	liver failure	43.0	WES	Com	NBAS	NBAS	_	c.2836T>C chr2:15555771 p.S946P<br>c.302dupT chr2:15693582 p.L101fs	missense mutation<br>insertion mutation	Father<br>Mother	YES<br>YES	Tolerated	Possibly damaging	Damaging	VUS<br>LP
88	F	B	NO	Liver dysfunciton	8.0	WES	Het	Negative	NBAS	YES	c.1648G>T chr2:15613423 p.G550C<br>c.6124A>G chr2:15374691 p.M2042V	missense mutation<br>missense mutation	Mother<br>Mother	YES<br>YES	Damaging<br>Tolerated	Benign<br>Benign	Damaging<br>Tolerated	VUS<br>VUS
89	M	B	YES	Genetic liver disease	10.0	TGPS	Com	CDG-IIb	MOGS		c.664G>A chr2:74690429 p.G222R<br>c.85C>G chr2:74692290 p.R29G	missense mutation<br>missense mutation	Father<br>Mother	YES<br>YES	Tolerated<br>Damaging	Benign<br>Benign	Damaging<br>Tolerated	VUS<br>VUS
90	F	B	NO	Liver dysfunciton	4.9	TGPS	Com	Negative	ACSF3	YES	c.356G>A chr16:89167445 p.G119D<br>c.661G>A chr16:89211764 p.A221T	missense mutation<br>missense mutation	Mother<br>Father	YES<br>YES	Damaging<br>Damaging	Possibly damaging<br>Possibly damaging	Damaging<br>Damaging	VUS<br>VUS
91	F	B	NO	Liver dysfunciton	28.0	TGPS	Het	OTCD	OTC	_	c.663+1G>A chrX:38262994 splicing	splicing mutation	Mother	NO	_	_	Damaging	P
92	M	B	NO	Wilson's disease	170.0	TGPS	Neg	Negative	_	_	_	_	_	_	_	_	_	_
93	F	B	NO	Liver dysfunciton	24.0	TGPS	Het	Negative	SLC25A13	YES	c.1661G>A chr7:95751243 p.R554Q	missense mutation	Father	NO	Damaging	Possibly damaging	Damaging	
94	F	B	NO	Liver dysfunciton	12.0	TGPS	Neg	Negative	_	_	_	_	_	_	_	_	_	_
95	M	B	NO	Reye's syndrome	31.0	TGPS	Neg	Negative	_	_	_	_	_	_	_	_	_	_
96	M	B	NO	Wilson's disease	120.0	TGPS	Het	Negative	ATP7B	YES	c.3443T>C chr13:52515330 p.I1148T	missense mutation	Father	NO	Damaging	Possibly damaging	Damaging	P

97	M	B	NO	Reye's syndrome	12.0	TGPS	Neg	Negative	_	_	_	_	_	_	_	_	_	_
98	F	B	NO	Liver dysfunciton	60.0	TGPS	Neg	Negative	_	_	_	_	_	_	_	_	_	_
99	F	B	NO	Liver dysfunciton	48.0	TGPS	Neg	Negative	_	_	_	_	_	_	_	_	_	_
100	F	B	NO	Liver dysfunciton	12.0	TGPS	Neg	Negative	_	_	_	_	_	_	_	_	_	_
101	M	B	NO	Genetic liver diseas	106.0	TGPS	Neg	Negative	_	_	_	_	_	_	_	_	_	_
102	F	B	NO	Genetic liver diseas	4.9	TGPS	Neg	Negative	_	_	_	_	_	_	_	_	_	_
103	F	B	NO	Genetic liver diseas	5.1	WES	Neg	Negative	_	_	_	_	_	_	_	_	_	_
104	M	B	NO	Liver dysfunciton	10.0	TGPS	Neg	Negative	_	_	_	_	_	_	_	_	_	_
105	M	B	NO	Wilson's disease	8.0	TGPS	Neg	Negative	_	_	_	_	_	_	_	_	_	_
106	M	B	NO	Liver dysfunciton	9.7	WES	Neg	Negative	_	_	_	_	_	_	_	_	_	_
107	M	B	NO	Genetic liver diseas	12.0	WES	Neg	Negative	_	_	_	_	_	_	_	_	_	_
108	F	B	NO	Liver dysfunciton	4.4	WES	Neg	Negative	_	_	_	_	_	_	_	_	_	_
109	F	B	NO	Genetic liver diseas	15.0	WES	Neg	Negative	_	_	_	_	_	_	_	_	_	_
110	F	B	NO	Wilson's disease	85.0	TGPS	Com	Wilson's disease	ATP7B	_	c.2810delT chr13-52523853*1 p.V937Gfs*5<br>c.2333G>T chr13-52532469*2 p.R778L	deletion mutation<br>missense mutation	Father<br>Mother	NO<br>NO	_<br>Damaging	–<br>Possibly damaging	_<br>Damaging	P<br>P
111	F	B	NO	Liver dysfunciton	15.0	WES	Neg	Negative	_	_	_	_	_	_	_	_	_	_
112	F	B	NO	Liver dysfunciton	14.0	WES	Neg	Negative	_	_	_	_	_	_	_	_	_	_
113	F	B	NO	Liver dysfunciton	36.0	WES	Het	Negative	COL6A2	YES	c.3026G>T chr21:47552432 p.G1009V	missense mutation	Father	NO	Tolerated	Benign	Damaging	VUS
114	M	B	NO	Liver dysfunciton	22.0	WES	Neg	Negative	_	_	_	_	_	_	_	_	_	_
115	M	B	NO	Genetic liver disease		WES	Het	Negative	AGL	YES	_	_	_	_	_	_	_	_
116	M	B	NO	Genetic liver diseas	96.0	WES	Het	AGS	IFIH1	_	c.2454+1G>C chr2:163130304 splicing	splicing mutation	de novo	YES	_	_	Damaging	P
117	F	B	NO	Genetic liver diseas	108.0	WES	Neg	Negative	_	_	_	_	_	_	_	_	_	
118	M	B	NO	Wilson's disease	42.0	TGPS	Het	Negative	ATP7B	YES	c.2447+5G>T chr13:52531647 splicing	splicing mutation	Mother	NO	_	_	_	LP

119	M	B	NO	Liver dysfunciton	2.0	WES	Het	Danon disease	LAMP2	_	c.928G>A chrX:119576454 p.V310I	missense mutation	Mother	NO	Tolerated	Benign	Tolerated	P
120	M	B	NO	Liver dysfunciton	4.4	WES	Het	Negative	ABCB4	YES	c.2860G>A chr7:87041273 p.G954S	missense mutation	Mother	NO	Damaging	Possibly damaging	Damaging	LP
121	F	B	NO	Liver dysfunciton	52.0	WES	Neg	Negative	_	_	_	_	_	_	_	_	_	_
122	M	B	NO	Liver dysfunciton	7.0	WES	Neg	Negative	_	_	_	_	_	_	_	_	_	_
123	M	B	NO	Genetic liver diseas	167.0	WES	Neg	Negative	_	_	_	_	_	_	_	_	_	_
124	F	B	NO	Genetic liver diseas	14.0	WES	Het	Joubert syndrome	APOB<br>OFD1	YES<br>_	c.4005C>G chr2:21236243 p.F1335L<br>c.8719C>T chr2-21231021 p.R2907C<br>c.223A>G chrX:13754708 p.N75D	missense mutation<br>missense mutation<br>missense mutation	Father<br>Father<br>Mother	YES<br>YES<br>YES	Damaging<br>Damaging<br>Damaging	Benign<br>Benign<br>Probabaly damaging	Tolerated<br>Tolerated<br>Damaging	VUS<br>VUS<br>VUS
125	F	B	NO	Liver dysfunciton	19.0	WES	Het	OTCD	OTC	_	c.532delG chrX:38260675 p.T178Rfs9	deletion mutation	denovo	YES	_	_	_	p
126	F	B	NO	Liver dysfunciton	2.9	WES	Neg	Negative	_	_	_	_	_	_	_	_	_	_
127	M	B	NO	Liver dysfunciton	9.0	WES	Neg	Negative	_	_	_	_	_	_	_	_	_	_
128	M	B	NO	Liver dysfunciton	22.0	WES	Neg	Negative	_	_	_	_	_	_	_	_	_	_
129	M	B	NO	Wilson's disease	33.0	WES	Com	Wilson's disease	ATP7B	_	c.2333G>T chr13:52532469 p.R778L<br>c.2576-6_2576-13delTTCCTACGinsAGGTGTCATGTC chr13:52524303-52524310 splicing	missense mutation<br>splicing mutation	Father<br>Mother	NO<br>YES	Damaging<br>_	Possibly damaging<br>_	Damaging<br>_	p<br>P
130	M	B	NO	Wilson's disease	38.0	WES	Com	Wilson's disease	ATP7B	_	c.2975C>T chr13:52520505 p.P992L<br>c.2621C>T chr13-52524252 p.A874V	missense mutation<br>missense mutation	Father<br>Mother	NO<br>NO	Damaging<br>Damaging	Possibly damaging<br>Possibly damaging	Damaging<br>Damaging	P<br>P
131	M	B	NO	Wilson's disease	182.0	WES	Neg	Negative	_	_	_	_	_	_	_	_	_	_
132	M	B	NO	Wilson's disease	108.0	TGPS	Com	Wilson's disease	ATP7B	_	c.2975C>T chr13:52520505 p.P992L<br>c.2333G>T chr13:52532469 p.R778L	missense mutation<br>missense mutation	Mother<br>Father	NO<br>NO	Damaging<br>Damaging	Possibly damaging<br>Possibly damaging	Damaging<br>Damaging	P<br>P
133	F	B	NO	Wilson's disease	115.0	WES	Hom	PFIC 3	ABCB4	_	c.431G>A chr7:87082365 p.R144Q	missense mutation	Mother Father	NO	Damaging	Possibly damaging	Damaging	LP
134	M	C	NO	Genetic liver diseas	5.0	WES	Neg	Negative	_	_								
135	F	C	NO	NPD	130.0	WES	Com	NPD	SMPD1	_	c.1101dupG chr11:6414449 p.F368Vfs*23<br>c.1517A>G chr11-6415458 p.Y506C	insertion mutation<br>missense mutation	Father<br>Mother	NO<br>YES	_<br>Damaging	_<br>Possibly damaging	_<br>Damaging	P<br>LP
136	F	C	NO	GSD	12.0	TGPS	Com	GSD VI	PYGL	_	c.1370G>A chr14:51381465 p.R457H<br>c.670+1G>A chr14:51387673 splicing	missense mutation<br>splicing mutation	Father<br>Mother	NO<br>YES	Damaging<br>_	Possibly damaging<br>_	Damaging<br>Damaging	LP<br>LP
137	F	C	NO	GSD	12.0	TGPS	Com	GSD Ia	G6PC	_	c.248G>A chr17:41055965 p.R83H<br>c.648G>T chr17:41063017 p.L216L	missense mutation<br>synonymous mutation	Father<br>Mother	NO<br>NO	Damaging<br>_	Possibly damaging<br>_	Damaging<br>_	P<br>P
138	F	C	NO	GSD	12.0	TGPS	Com	GSD Ia	G6PC	_	c.260delG chr17:41055977 p.V88Ffs*14<br>c.1022T>A chr17:41063391 p.I341N	deletion mutation<br>missense mutation	Father<br>Mother	NO<br>NO	_<br>Damaging	_<br>Possibly damaging	_<br>Damaging	P<br>P

139	M	C	NO	GSD	6.8	TGPS	Com	GSD Ia	G6PC	_	c.248G>A chr17:41055965 p.R83H<br>c.648G>T chr17:41063017 p.L216L	missense mutation<br>synonymous mutation	Father<br>Mother	NO<br>NO	Damaging<br>_	Possibly damaging<br>_	Damaging<br>_	P<br>P
140	M	C	NO	GSD	11.6	TGPS	Com	GSD Ia	G6PC	_	c.247C>T chr17:41055964 p.R83C<br>c.648G>T chr17:41063017 p.L216L	missense mutation<br>synonymous mutation	Mother<br>Father	NO<br>NO	Damaging<br>_	Possibly damaging<br>_	Damaging<br>_	P<br>P
141	M	C	NO	GSD	22.0	TGPS	Het	GSD IXa	PHKA2	_	c.3614C>T chrX:18911697 p.P1205L	missense mutation	Mother	NO	Damaging	Possibly damaging	Damaging	P
142	F	C	NO	GSD	4.9	TGPS	Com	GSD Ia	G6PC	_	c.248G>A chr17:41055965 p.R83H<br>c.648G>T chr17:41063017 p.L216L	missense mutation<br>synonymous mutation	Father<br>Mother	NO<br>NO	Damaging<br>_	Possibly damaging<br>_	Damaging<br>_	P<br>P
143	M	C	NO	GSD	36.0	TGPS	Com	GSD VI	PYGL	_	c.2426C>T chr14:51372228 p.S809L<br>c.280C>T chr14:51404519 p.R94*	missense mutation<br>nonsense mutation	Mother<br>Father	YES<br>NO	Tolerated<br>_	Possibly damaging<br>_	Damaging<br>Damaging	VUS<br>P
144	F	C	NO	GSD	23.0	TGPS	Com	GSD III	AGL	_	c.664+1G>A chr1:100330146 splicing<br>c.1735+1G>T chr1:100345603 splicing	splicing mutation<br>splicing mutation	Mother<br>Father	NO<br>NO	_<br>_	_<br>_	_<br>_	P<br>P
145	M	C	NO	GSD	13.0	WES	Com	GSD Ia	G6PC	_	c.648G>T chr17:41063017 p.L216L	synonymous mutation	Mother Father	NO	_	_	_	p
146	M	C	NO	GSD	57.0	WES	Hom	GSD Ia	G6PC	_	c.648G>T chr17:41063017 p.L216L	synonymous mutation	Mother Father	NO	_	_	_	P
147	M	C	NO	GSD	21.0	TGPS	Com	GSD	G6PC	_	c.648G>T chr17:41063017 p.L216L<br>c.674T>C chr17:41063043 p.L225P<br>chrM:11778G>A	synonymous mutation<br>missense mutation	Mother<br>Father<br>_	NO<br>NO<br>_	_<br>Tolerated<br>_	_<br>Possibly damaging<br>_	_<br>Damaging<br>_	P<br>VUS<br>P
148	M	C	NO	GSD	12.0	WES	Het	GSD IXa	PHKA2	_	c.883C>T chrX:18958148 p.R295C	missense mutation	Mother	NO	Damaging	Possibly damaging	Damaging	p
149	F	C	NO	GSD	12.0	WES	Com	GSD Ia	G6PC	_	c.648G>T chr17:41063017 p.L216L<br>c.508C>T chr17:41061381 p.R170*	synonymous mutation<br>nonsense mutation	Father	NO	_	_	_<br>Damaging	p<br>P
150	F	C	NO	GSD	28.0	WES	Hom	GSD Ia	G6PC	_	c.648G>T chr17:41063017 p.L216L	synonymous mutation	Mother Father	NO	_	_	_	P
151	F	C	NO	GSD	4.6	WES	Hom	GSD Ia	G6PC	_	c.248G>A chr17:41055965 p.R83H	missense mutation	Mother Father	NO	Damaging	Possibly damaging	Damaging	P
152	M	C	NO	GSD	67.0	WES	Het	GSD IXa	PHKA2	_	c.3341C>T chrX-18912518 p.T1114I	missense mutation	Mother	YES	Damaging	Possibly damaging	Damaging	P
153	M	C	NO	GSD	24.0	WES	Het	GSD IXa	PHKA2	_	c.1460-3C>G chrX：18943898 splicing	splicing mutation	Mother	YES	_	_	_	VUS
154	M	C	NO	GSD	34.0	TGPS	Het	GSD IXa	PHKA2	_	c.3275T>G chrX:18915288 p.L1092R	missense mutation	Mother	YES	Damaging	Possibly damaging	Damaging	VUS
155	F	C	NO	GSD	25.0	TGPS	Com	GSD III	AGL	_	c.3275T>G chr1:100340986 p.T420P<br>c.2929C>T chr1:100356892 p.R977*	missense mutation<br>nonsense mutation	Mother<br>Father	YES<br>NO	Damaging<br>_	Possibly damaging<br>_	Damaging<br>Damaging	LP<br>P
156	F	C	NO	GSD	30.0	WES	Hom	GSD III	AGL	_	c.4260-12A>G chr1:100381954 splicing	splicing mutation	Mother Father	NO	_	_	_	p
157	F	C	NO	NPD	51.0	TGPS	Com	NPD	SMPD1	_	c.668G>A chr11:6412963 p.C223Y<br>c.1486+5G>C chr11:6415276 splicing	missense mutation<br>splicing mutation	Mother<br>Father	YES<br>NO	Damaging<br>_	Possibly damaging<br>_	Damaging<br>_	VUS<br>VUS
158	M	C	NO	Lipid metabolitic disoder	12.0	TGPS	Hom	GD	GBA	_	c.1187T>C chr1:155205043 p.L396P	missense mutation	Mother Father	NO	Damaging	Possibly damaging	Damaging	p

159	F	C	NO	GSD	12.0	TGPS	Com	GSD VI	PYGL	_	c.1370G>A<br>chr14:51381465<br>p.R457H<br>c.670+1G>A<br>chr14:51387673<br>splicing	missense mutation<br>splicing mutation	Father<br>Mother	NO<br>YES	Damaging<br>_	Possibly damaging<br>_	Damaging<br>_	LP<br>LP
160	M	C	NO	acid metabolitic di	11.4	TGPS	Hom	VLCAD	ACADVL	_	c.844G>C<br>chr17:7126017<br>p.A282P	missense mutation	Mother<br>Father	NO	Damaging	Possibly damaging	Damaging	LP
161	F	C	NO	Gaucher disease	3.6	TGPS	Hom	GD	GBA	_	c.1187T>C<br>chr1:155205043<br>p.L396P	missense mutation	Mother<br>Father	NO	Damaging	Possibly damaging	Damaging	P
162	F	C	NO	GSD	4.2	TGPS	Hom	Transient infantile<br>hypertriglyceridemia	GPD1	_	c.454C>T<br>chr12:50500611<br>p.Q152*	missense mutation	Mother<br>Father	NO	_	_	Damaging	p
163	F	C	NO	Gaucher disease	8.3	TGPS	Het	Negative	GBA	YES	c.1342G>C<br>chr1:155205518<br>p.D448H	missense mutation	Mother	NO	Tolerated	Benign	Damaging	P
164	M	C	NO	NPD	12.0	TGPS	Neg	Negative	_	_	_	_	_	_	_	_	_	_
165	F	C	NO	Genetic liver diseas	45.0	WES	Hom	MPS 3B	NAGLU	_	c.1004A>G<br>chr17:40693207<br>p.Y335C	missense mutation	Father<br>Mother	NO	Damaging	Possibly damaging	Damaging	VUS
166	M	C	NO	GSD	12.0	TGPS	Het	Negative	GALK1	YES	c.593C>T<br>chr17:73759113<br>p.A198V	missense mutation	Mother	NO	Damaging	Benign	Damaging	VUS
167	M	C	NO	GSD	24.0	WES	Neg	Negative	_	_	_	_	_	_	_	_	_	_
168	F	C	YES	GSD	19.0	WES	Com	GSD III	AGL	_	c.1222C>T<br>chr1:100340950<br>p.R408*<br>c.1735+1G>T<br>chr1:100345603<br>splicing	nonsense mutation<br>splicing mutation	Mother<br>Not validated	NO<br>NO	_<br>_	_<br>_	Damaging<br>_	P<br>P
169	M	C	NO	GSD	18.0	WES	Neg	Negative	_	_	_	_	_	_	_	_	_	_
170	M	C	NO	Genetic liver diseas	38.0	WES	Het	GSD IXa1	PHKA2	_	c.2746C>T<br>chrX:18924673<br>p.R916W	missense mutation	Mother	NO	Damaging	Possibly damaging	Damaging	VUS
171	F	C	NO	GSD	9.5	TGPS	Hom	GSD Ia	G6PC	_	c.648G>T<br>chr17:41063017<br>p.L216L	shifting mutation	Mother<br>Father	NO	_	_	_	P
172	M	C	NO	GSD	21.0	WES	Het	GSD IXa1	PHKA2	_	c.401A>G<br>chrX:18969275<br>p.Q134R	missense mutation	Mother	NO	Damaging	Possibly damaging	Damaging	VUS
